# Supplementary material for: Phloroglucinol-Based Carbon Quantum Dots/Polyurethane Composite Films: How Structure of Carbon Quantum Dots Affects Antibacterial and Antibiofouling Efficiency of Composite Films
Source: Polymers (Basel). 2024 Jun 11;16(12):1646. doi: 10.3390/polym16121646 (PMC11207477; doi:10.3390/polym16121646)
Supplement: Supplementary file 1 [file polymers-16-01646-s001.zip › polymers-3021764-supplementary.pdf]

Supporting information

Phloroglucinol-Based Carbon Quantum Dots/Polyurethane Composite Films: How Structure of Carbon Quantum Dots Affects Antibacterial and Antibiofouling Efficiency of Composite Films

Zoran M. Marković [1,*], Dušan D. Milivojević [1], Janez Kovač [2] and Biljana M. Todorović Marković [1,*]

[1] Vinča Institute of Nuclear Sciences, National Institute of the Republic of Serbia, University of Belgrade, 11158 Belgrade, Serbia; dusanm@vinca.rs
[2] Department of Surface Engineering, Jozef Stefan Institute, Jamova 39, SI-1000 Ljubljana, Slovenia; janez.kovac@ijs.si
* Correspondence: zoranmarkovic@vin.bg.ac.rs (Z.M.M.); biljatod@vin.bg.ac.rs (B.M.T.M.); Tel.: +381-11-3408582 (Z.M.M.)

Table S1. Fitted PL spectra to 2 Gaussian peaks of PHL-CQDs nanoparticles and corresponding FWHMs.

	P1		P2		FWHM 1		FWHM 2	
λ (nm)	P11	P12	P21	P22	FWHM11	FWHM12	FWHM21	FWHM22
325	398.87	425.47	451.14	486.11	39.71	31.33	51.68	121.92
350	400.19	424.32	451.50	485.72	15.47	28.45	40.25	92.88
375	400.69	424.75	451.87	486.08	11.65	23.13	37.36	89.27
400	425.21	455.92	491.75	539.67	18.67	39.80	80.59	71.87
425		466.16	496.11	534.40		25.03	50.54	83.75
450		497.17		540.35		62.19		87.90
475		499.03		534.85		22.22		115.43
500		600.82		542.43		56.02		93.18
